# Nitrogenase Cofactor Maturase NifB Isolated from Transgenic Rice is Active in FeMo-co Synthesis

**DOI:** 10.1021/acssynbio.2c00194

**Published:** 2022-08-23

**Authors:** Wenshu He, Stefan Burén, Can Baysal, Xi Jiang, Teresa Capell, Paul Christou, Luis M. Rubio

**Affiliations:** † Department of Plant Production and Forestry Science, University of Lleida-Agrotecnio CERCA Center, Av. Alcalde Rovira Roure, 191, 25198 Lleida, Spain; ‡ Centro de Biotecnología y Genómica de Plantas, Universidad Politécnica de Madrid (UPM), Instituto Nacional de Investigación y Tecnología Agraria y Alimentaria (INIA), Campus Montegancedo UPM, Pozuelo de Alarcón, 28223 , Madrid, Spain; § Departamento de Biotecnología-Biología Vegetal, Escuela Técnica Superior de Ingeniería Agronómica, Alimentaria y de Biosistemas, Universidad Politécnica de Madrid, 28040 Madrid, Spain; ∥ ICREA, Catalan Institute for Research and Advanced Studies, Passeig Lluís Companys 23, 08010 Barcelona, Spain

**Keywords:** nitrogen fixation, transgenic rice, NifB-co, synthetic biology, iron-molybdenum cofactor, iron-sulfur cluster

## Abstract

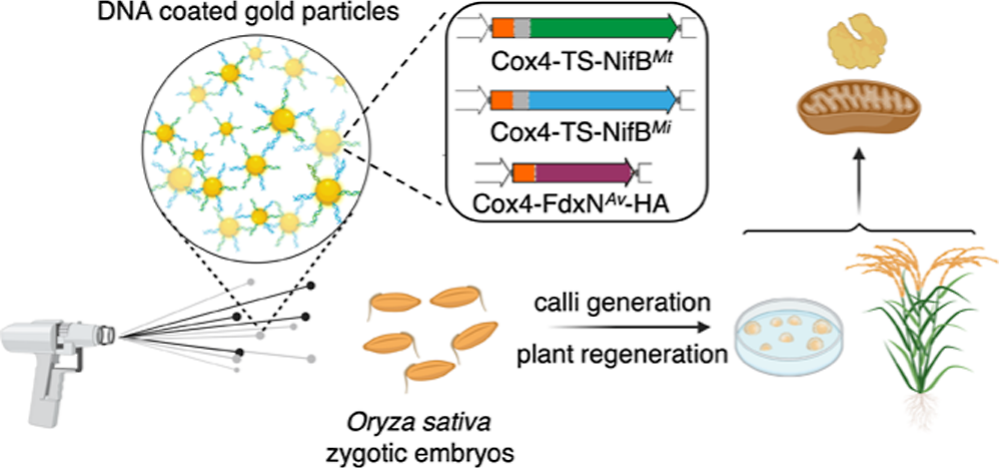

The engineering of nitrogen fixation in plants requires assembly of an active prokaryotic nitrogenase complex, which is yet to be achieved. Nitrogenase biogenesis relies on NifB, which catalyzes the formation of the [8Fe–9S–C] metal cluster NifB-co. This is the first committed step in the biosynthesis of the iron–molybdenum cofactor (FeMo-co) found at the nitrogenase active site. The production of NifB in plants is challenging because this protein is often insoluble in eukaryotic cells, and its [Fe–S] clusters are extremely unstable and sensitive to O_2_. As a first step to address this challenge, we generated transgenic rice plants expressing NifB from the Archaea *Methanocaldococcus infernus* and *Methanothermobacter thermautotrophicus*. The recombinant proteins were targeted to the mitochondria to limit exposure to O_2_ and to have access to essential [4Fe–4S] clusters required for NifB-co biosynthesis. *M. infernus* and *M. thermautotrophicus* NifB accumulated as soluble proteins *in planta*, and the purified proteins were functional in the in vitro FeMo-co synthesis assay. We thus report NifB protein expression and purification from an engineered staple crop, representing a first step in the biosynthesis of a functional NifDK complex, as required for independent biological nitrogen fixation in cereals.

## Introduction

Nitrogen (N) fertilizers are a major input to sustain high yields of cereal crops.^1^ However, high use of N fertilizers has negative impact on human health and the environment.^2,3^ Engineering biological nitrogen fixation (BNF) in cereals is necessary to reduce our dependency on N fertilizers.^4^ BNF involves the conversion of inert atmospheric N_2_ into biologically useable ammonia catalyzed by the enzyme nitrogenase. However, nitrogenases are only found in some bacteria and archaea, so plants cannot fix their own nitrogen and must either form symbiotic relationships with nitrogen-fixing prokaryotes or obtain fixed nitrogen compounds from the soil.^5^ One potential strategy to develop nitrogen-fixing cereal crops is by engineering the transfer of bacterial *ni*trogen *f*ixation (*nif*) genes to the plant genome,^6,7^ but this process requires deep understanding of nitrogenase activity requirements and performance in eukaryotic cells.

There are three types of nitrogenases: molybdenum- (Mo), vanadium- (V), and iron-only (Fe) nitrogenases.^8^ The Mo-nitrogenase, the most widespread and studied one, consists of two metalloproteins: MoFe protein and Fe protein. The MoFe protein is a dinitrogenase encoded by *nifDK* that contains the catalytic site which binds and reduces N_2_. The Fe protein is a dinitrogenase reductase encoded by *nifH*, which provides electrons to the MoFe protein.^9^ Mo-nitrogenase has three essential metal clusters. The first is the [4Fe–4S] cluster located in the Fe protein, and the others are the P-cluster [8Fe–7S] and FeMo-co [7Fe–9S–C–Mo-*R*-homocitrate] located in the MoFe protein.^10^

The [4Fe–4S] clusters are synthesized by NifU and NifS, where NifU provides the scaffold for the assembly of [Fe–S] clusters, and NifS mobilizes sulfur (S) from cysteine for [Fe–S] cluster synthesis on NifU.^11,12^ The P-cluster is formed by the reductive coupling of two [4Fe–4S] cluster pairs at the MoFe protein in the presence of the Fe protein, MgATP, and a reductant.^13,14^ FeMo-co is one of the most complex [Fe–S] clusters discovered in nature thus far and is synthesized in a regulated and coordinated process, depending on a multitude of proteins.

The proteins involved in FeMo-co biosynthesis can be functionally divided into four classes: molecular scaffolds (NifU, NifB, and NifEN), metallocluster carriers (NifX, NafY, and NifY), substrate providers (NifS, NifQ, and NifV), and NifH.^15^ The process of FeMo-co biosynthesis is initiated by NifU and NifS with the assembly of [4Fe–4S] cluster units that are transferred to NifB.^16−18^ NifB is an *S*-adenosyl-l-methionine (SAM) radical enzyme that carries a catalytic [4Fe–4S] cluster (called RS cluster) and two additional [4Fe–4S] clusters (called K1- and K2-clusters) used as substrates to generate the [8Fe–9S–C] product called NifB-co.^19−22^ It has been shown that NifB-co production is 80% lower in *Azotobacter vinelandiifdxN* mutants, suggesting that FdxN is required for NifB-co biosynthesis.^23^ Although the precise function of FdxN remains unclear, it is thought to provide NifB with electrons needed for NifB-co formation.^10^ NifB-co synthesized by NifB is matured into FeMo-co on NifEN/NifH complexes,^24,25^ which is then inserted into the active site of the apo-MoFe protein to reconstitute active Mo-nitrogenase.^26^ As the function of NifB is to catalyze the first committed step in the biosynthesis of FeMo-co (the production of NifB-co), *nifB* mutants lack FeMo-co.^27,28^ NifB-co is also the precursor cluster for the biosynthesis of FeV-co and FeFe-co found at the active sites of the alternative V-nitrogenase and Fe-only nitrogenase^17,29^ and therefore required for all BNF.

The NifB proteins of *A. vinelandii* and *Klebsiella oxytoca* comprise an N-terminal SAM-radical domain containing the CxxxCxxC SAM-binding motif and a C-terminal NifX-like domain.^30,31^ However, the simplest NifB architecture is a standalone SAM-radical domain because the NifX-like sequence is not essential for NifB activity. For example, the single-domain NifB proteins from *Methanosarcina acetivorans*, *Methanobacterium thermoautotrophicum*, *Methanocaldococcus infernus*, and *Methanothrix thermoacetophila* are functionally equivalent to *A. vinelandii* NifB.^19,30,32^ This single-domain architecture facilitates the heterologous expression of stable NifB in *Escherichia coli*.^32,33^ When a library of 28 NifB proteins was screened for expression in *Saccharomyces cerevisiae,* only six accumulated as predominantly soluble proteins targeted to mitochondria, and four of these had a single-domain architecture.^34^ When the same NifB variants were expressed transiently in *Nicotiana benthamiana* and targeted to the mitochondria or chloroplasts, only three accumulated as soluble proteins. All three were single-domain proteins, corroborating their superior expression and performance in eukaryotic hosts.^35^

Here, we generated rice plants expressing stably *M. infernus* NifB or *Methanothermobacter thermautotrophicus* NifB targeted to the mitochondria, in each case, together with *A. vinelandii* FdxN. Both NifB proteins accumulated in a soluble form, and their functionality was confirmed by FeMo-co synthesis in vitro.*M. thermautotrophicus* NifB exhibited higher NifB-co conversion activity in vitro, compared with*M. infernus* NifB. The production of functional NifB in rice represents an important step toward the expression of active nitrogenase to achieve BNF in cereal crops.

## Results

### Genetic Elements and Rice Transformation

*S. cerevisiae* codon-optimized *nifB* from *M. infernus* (*nifB*^*Mi*^) and *M. thermautotrophicus* (*nifB*^*Mt*^) and *fdxN* from *A. vinelandii* (*fdxN*^*Av*^) were used for rice transformation because there are no rare codons in these three gene sequences in the context of rice codon usage.^36^ The *nifB*^*Mi*^ gene (hereafter *OsnifB*^*Mi*^ to indicate expression in rice), *nifB*^*Mt*^ (hereafter *OsnifB*^*Mt*^), and the *fdxN*^*Av*^ gene (hereafter *OsfdxN*^*Av*^) were introduced into separate vectors for rice transformation. Expression was driven by the strong constitutive *ZmUbi1* + *1st i* promoter. An N-terminal mitochondrial leader sequence from the *S. cerevisiae* cytochrome c oxidase subunit IV (Cox4) was added to direct the proteins to mitochondria because this sequence was previously shown to target recombinant eGFP to the rice mitochondria effectively.^37^ An N-terminal Twin-Strep (TS) tag was added between the Cox4 signal and the *Os*NifB^*Mi*^ or *Os*NifB^*Mt*^ proteins for detection and purification of *Os*NifB. A C-terminal hemagglutinin (HA) tag was added to *Os*FdxN^*Av*^ to enable immunodetection. The *OsnifB*^*Mi*^ and *OsnifB*^*Mt*^ constructs were used separately, in each case, combined with *OsfdxN*^*Av*^ and a third construct carrying the hygromycin phosphotransferase (*hpt*) gene for selection. Transgenic rice callus expressing *nif* transgenes were produced by direct DNA transfer, as described.^38,39^ Plantlets were regenerated from the corresponding callus lines under hygromycin selection and grown to maturity, as described.^38,39^

### Transgenic Rice Callus and Recovery of Plants Expressing *OsnifB* and *OsfdxN*

**Figure 1:**
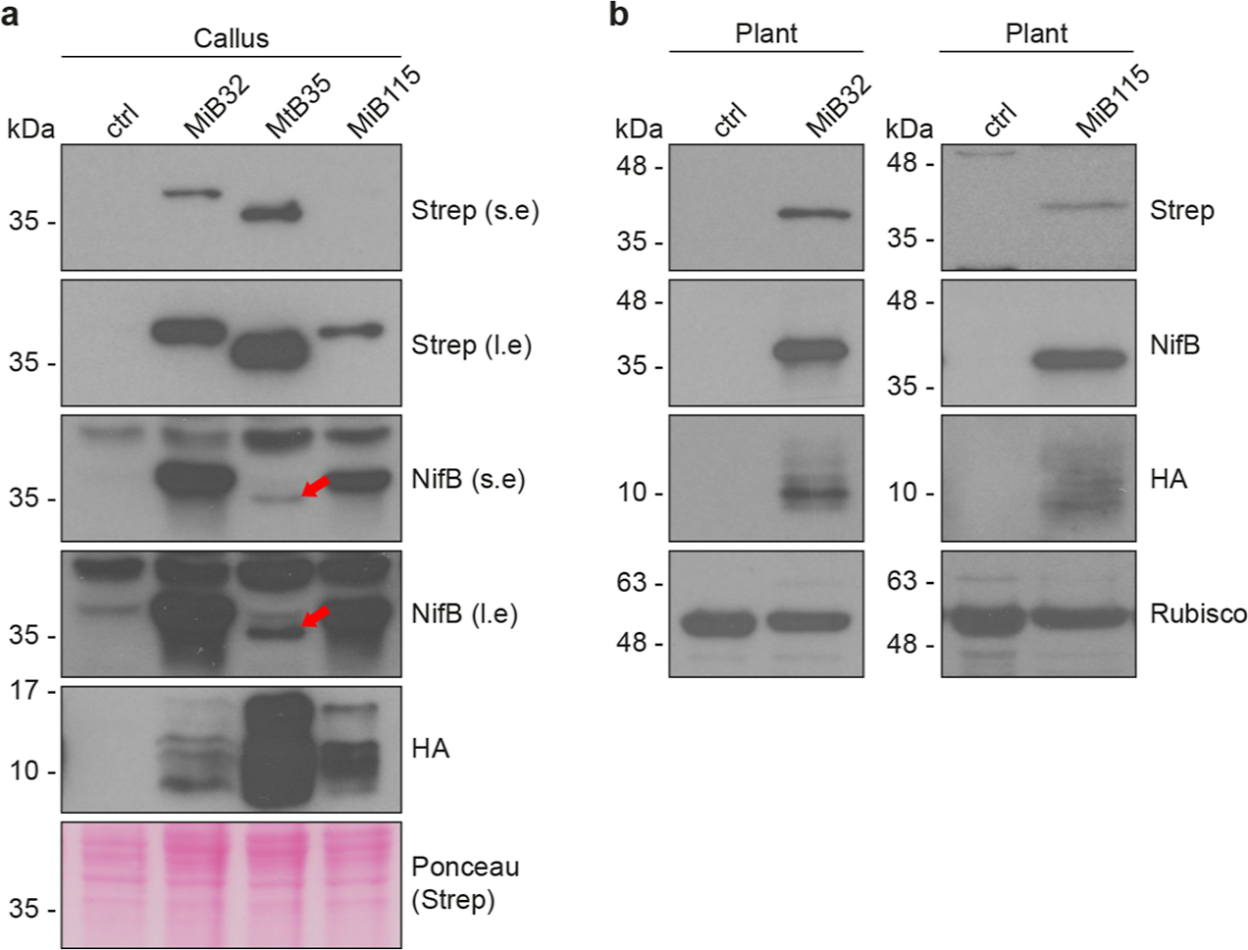
Expression of *Os*NifB^*Mi*^, *Os*NifB^*Mt*^, and *Os*FdxN^*Av*^ in callus and plants. Immunoblot analysis of cell-free extracts prepared from callus (a) and plants (b). *Os*NifB^*Mi*^ and *Os*NifB^*Mt*^ were detected with antibodies against NifB^*Mi*^ and the N-terminal TS tag. *Os*FdxN^*Av*^ was detected with antibodies against the C-terminal HA tag. The red arrow indicates the signal from *Os*NifB^*Mt*^ detected with antibodies against NifB^*Mi*^. Abbreviations: *Os*NifB^*Mi*^: *O. sativa*-derived *M. infernus* NifB; *Os*NifB^*Mt*^: *O. sativa*-derived *M. thermautotrophicus* NifB; *Os*FdxN^*Av*^: *O. sativa*-derived *A. vinelandii* FdxN; s.e.: short exposure during immunoblot detection; l.e.: long exposure during immunoblot detection; MiB32, MiB115, and MtB35 are three independent lines. N.B. *Os*NifB^*Mt*^ was not detectable in multiple regenerated siblings from line MtB35.

We recovered transgenic lines co-expressing NifB and FdxN at the mRNA level (Figure S1). Four lines each from MiB and MtB were selected for immunoblot analysis. We identified three lines, MiB32, MiB115, and MtB35 that accumulated the recombinant proteins at the highest levels. Accumulation of *Os*NifB^*Mi*^, *Os*NifB^*Mt*^, and *Os*FdxN^*Av*^ in these callus lines and the corresponding regenerated plants was determined by immunoblot analysis using antibodies specific for NifB^*Mi*^, and the TS and HA tags (Figures 1, S2, and S3). Based on their SDS-gel migration patterns, the recombinant proteins were correctly processed, resulting in the expected molecular weights of 38 kDa (*Os*NifB^*Mi*^), 35 kDa (*Os*NifB^*Mt*^), and 11 kDa (*Os*FdxN^*Av*^). As seen previously when expressed in *S. cerevisiae*,^34,40^ the migration of the HA-tagged FdxN^*Av*^ protein was less distinct, probably due to its smaller size. We thus confirmed that *Os*NifB^*Mi*^, *Os*NifB^*Mt*^, and *Os*FdxN^*Av*^ proteins accumulated in the soluble form in rice callus (Figure 1a). *Os*NifB^*Mi*^ and *Os*FdxN^*Av*^ were soluble in regenerated plants (Figure 1b). We were not able to detect accumulation of *Os*NifB^*Mt*^ in regenerated MtB35 plants.

### Purification of *Os*NifB^*Mi*^ and *Os*NifB^*Mt*^

**Figure 2:**
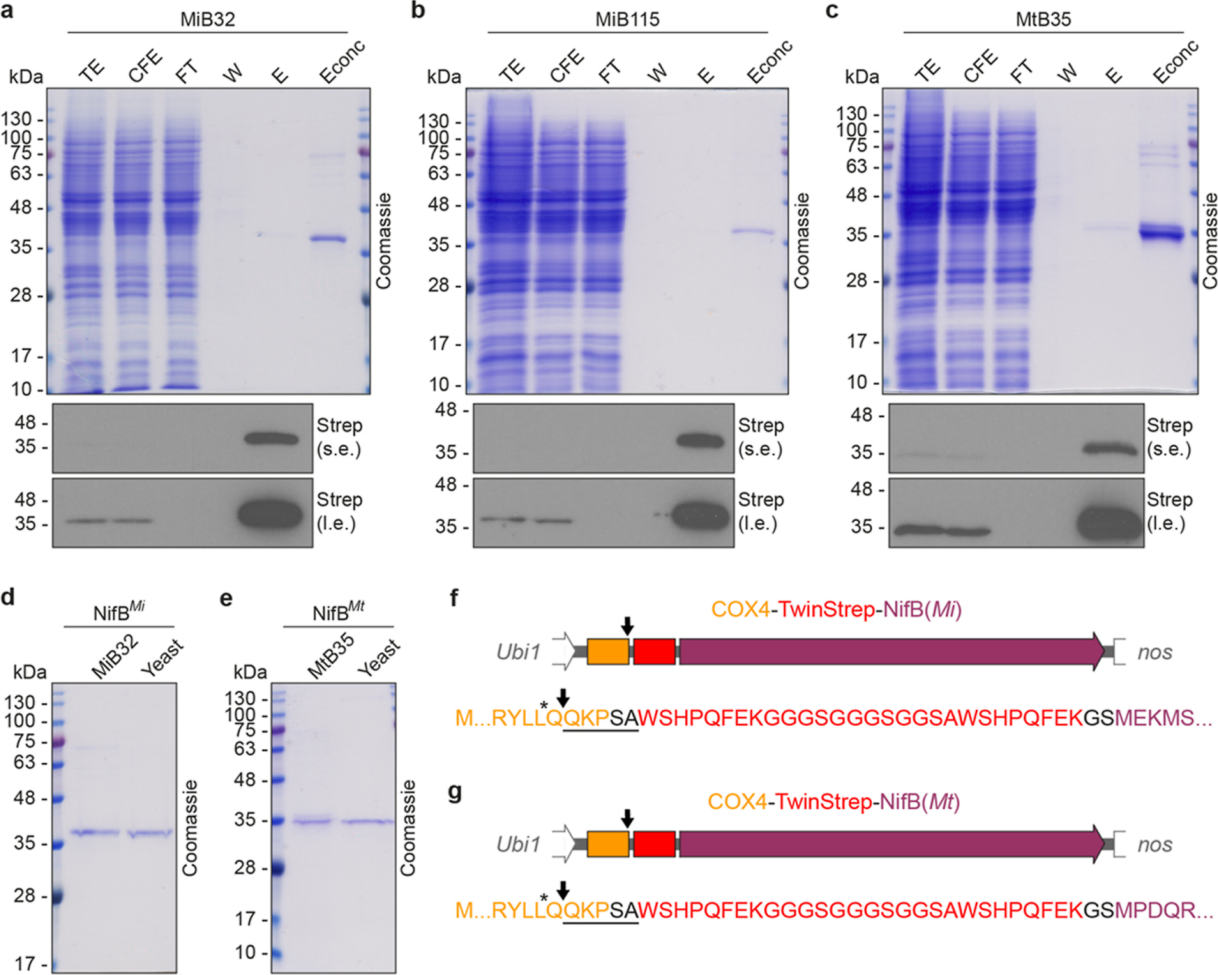
STAC purification and N-terminal sequence of *Os*NifB^*Mi*^ and *Os*NifB^*Mt*^. Purification of *Os*NifB from MiB32 (a), MiB115 (b), and MtB35 (c) callus. TE: total extract, CFE: soluble cell-free extract, FT: flow-through fraction, W: wash fraction, and E: elution fraction. Fractions were analyzed by SDS-PAGE, followed by Coomassie gel staining or immunoblot analysis using antibodies detecting the TS tag. Migration of STAC-purified *Os*NifB^*Mi*^ (MiB32) and *Sc*NifB^*Mi*^ (d) and *Os*NifB^*Mt*^ (MtB35) and *Sc*NifB^*Mt*^ (e). Cleavage sites of Cox4 in *Os*NifB^*Mi*^ (f) and *Os*NifB^*Mt*^ (g). The black arrow indicates the N-terminal processing site as determined by N-terminal sequencing. The underlined amino acids represent those detected by the Edman degradation procedure. The black stars indicate the cleavage site for endogenous Cox4 in *S. cerevisiae*.

The *Os*NifB^*Mi*^ and *Os*NifB^*Mt*^ proteins were purified from rice callus lines (MiB32, MiB115, and MtB35) by strep-tag affinity chromatography (STAC), and the purification process was monitored by sampling the total extract, cell-free extract, flow-through, wash, and elution fractions for analysis by SDS-PAGE and immunoblotting (Figures 2a–c and S4). No significant amount of protein was lost during centrifugation and filteration of the cell extract, confirming that both *Os*NifB^*Mi*^ and *Os*NifB^*Mt*^ proteins were soluble in the mitochondria. The elution fractions featured a band matching the anticipated size of correctly processed *Os*NifB^*Mi*^ or *Os*NifB^*Mt*^. Isolated NifB yields were 44 and 87 μg per 100 g fresh weight callus for *Os*NifB^*Mi*^ and *Os*NifB^*Mt*^, respectively (Figure S5). Side by side comparison of NifB^*Mi*^ and NifB^*Mt*^ proteins isolated from yeast and rice suggested the correct targeting and the specific processing of Cox4 signals from both *Os*NifB proteins (Figure 2d,e).^34^ The exact cleavage site of the Cox4 sequence was further investigated by N-terminal sequencing. Cleavage was specific after amino acid 26 in the Cox4 peptide (Figure 2f,g), which is only one amino acid away from where the endogenous Cox4 protein is processed in *S. cerevisiae*,^41^ generating a single *Os*NifB protein moiety. Removal of the Cox4 signal, following the import of *Os*NifB^*Mi*^ and *Os*NifB^*Mt*^ to the mitochondria, therefore confirmed successful targeting.

### *Os*NifB^*Mi*^ and *Os*NifB^*Mt*^ Catalyzes FeMo-co Synthesis In Vitro

The minimal protein components for FeMo-co synthesis in vitro are NifB, NifEN, and NifH.^26^ The isolated *Os*NifB^*Mi*^ or *Os*NifB^*Mt*^ protein was mixed with [4Fe–4S] cluster-loaded *A. vinelandii* NifU purified from *E. coli* (*Ec*NifU^*Av*^, as the source of [4Fe–4S] precursor clusters for NifB-co biosynthesis), *A. vinelandii* NifEN with the permanent [4Fe–4S] clusters but lacking FeMo-co precursor cluster (apo-NifEN^*Av*^), *A. vinelandii* NifH protein (NifH^*Av*^), and *A. vinelandii* NifDK with the P-cluster but lacking FeMo-co (apo-NifDK^*Av*^). Molybdate, *R*-homocitrate, and SAM were added as they are the required substrates for NifB-dependent in vitro FeMo-co synthesis.

**Figure 3:**
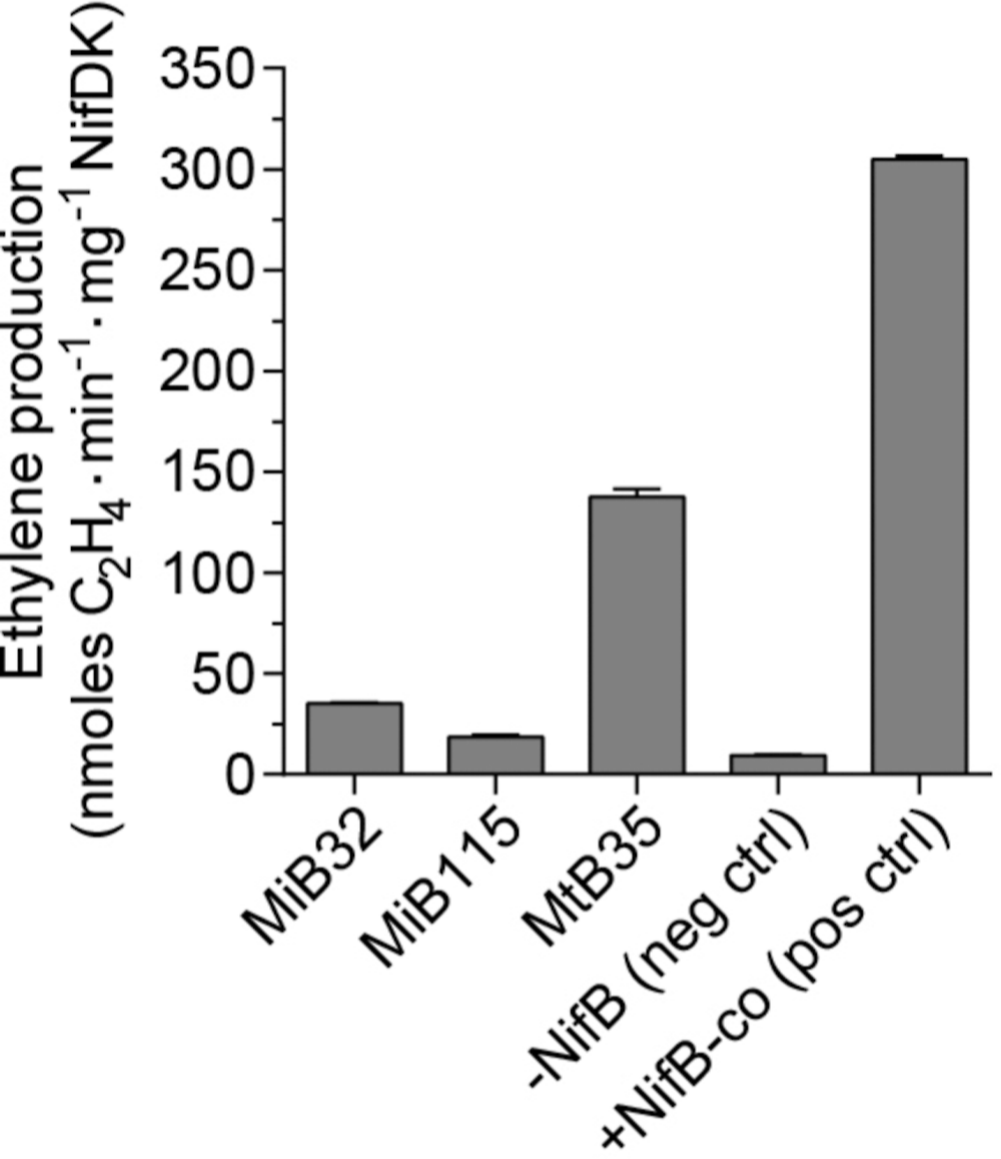
In vitro FeMo-co synthesis and apo-NifDK reconstitution using the as-isolated *Os*NifB^*Mi*^ and *Os*NifB^*Mt*^ proteins supplemented with [4Fe–4S] cluster substrates. Activity is represented as nanomoles of ethylene produced per minute and milligram of NifDK. The activity of the positive control reaction for FeMo-co synthesis (containing pure NifB-co instead of NifB) was 305 ± 2 units, and the activity of the ATP-mix control reaction (containing holo-NifDK) was 1506 ± 95 units. MiB32, MiB115, and MtB35 denote *Os*NifB^*Mi*^ or *Os*NifB^*Mt*^ isolated from three independent lines. Data are means ± SD (*n* = 2).

The as-isolated *Os*NifB^*Mi*^ and *Os*NifB^*Mt*^ proteins were colorless and inactive in FeMo-co synthesis. However, when loaded with [4Fe–4S] clusters from NifU, the *Os*NifB^*Mi*^ and *Os*NifB^*Mt*^ proteins were functional in the FeMo-co synthesis assay, in which the *Os*NifB-dependent activation of apo-NifDK was measured by in vitro acetylene reduction activity of the reconstituted enzyme (Figure 3). This shows that the NifB-co produced by *Os*NifB matured into FeMo-co at the NifEN/NifH complex, which was then transferred to apo-NifDK^*Av*^. The OsNifB^*Mi*^-dependent activation of apo-NifDK resulted in nitrogenase activities of 35 ± 0.95 and 19 ± 0.94 nmol C_2_H_4_ min^–1^ mg^–1^ NifDK using *Os*NifB^*Mi*^ isolated from lines MiB32 and MiB115, respectively, while FeMo-co synthesis using OsNifB^*Mt*^ isolated from MtB35 resulted in fourfold higher nitrogenase activities (137 nmol C_2_H_4_ min^–1^ mg^–1^ NifDK) (Figure 3).

**Figure 4:**
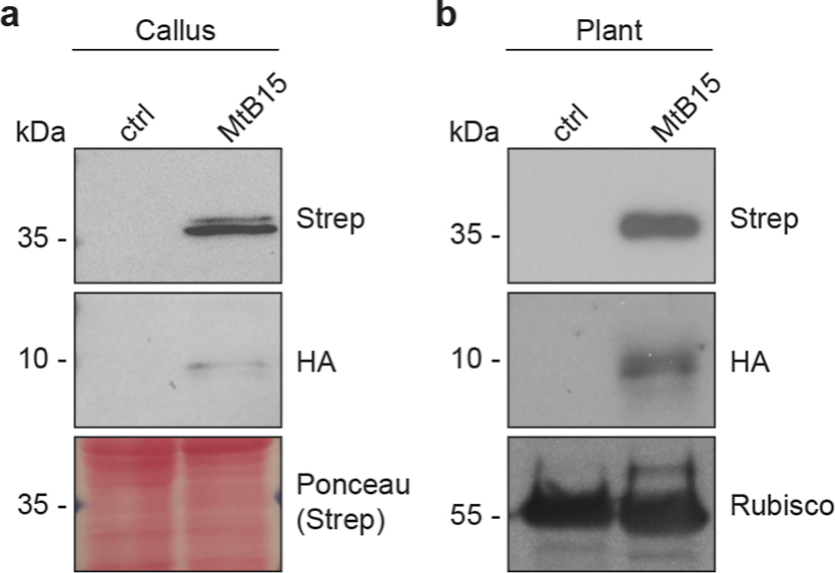
Accumulation of *Os*NifB^*Mt*^ and *Os*FdxN^*Av*^ in rice callus (a) and plants (b). *Os*NifB^*Mt*^ was detected with antibodies against the N-terminal TS tag. *Os*FdxN^*Av*^ was detected with antibodies against the C-terminal HA tag. Abbreviations: *Os*NifB^*Mt*^: *O. sativa*-derived *M. thermautotrophicus* NifB; *Os*FdxN^*Av*^: *O. sativa*-derived *A. vinelandii* FdxN. MtB15 is a line accumulating *Os*NifB^*Mt*^ in callus and leaves.

**Figure 5:**
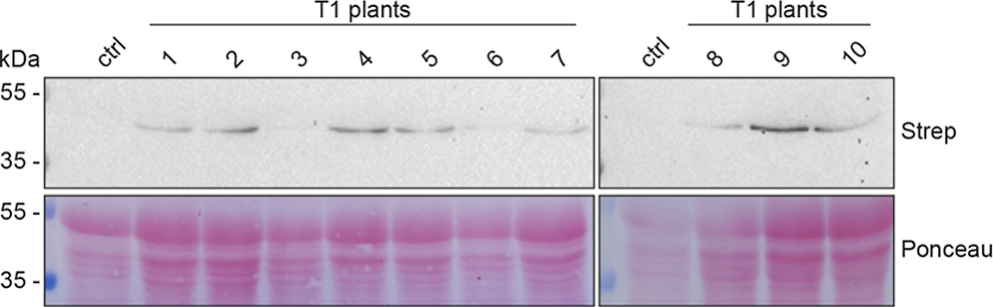
Expression of *Os*NifB^*Mi*^ in 10 different T1 plants from the MiB115 line. Immunoblot analysis was performed using leaf soluble protein extracts and antibodies detecting the TS tag. The control lane was loaded with leaf soluble protein extracts obtained from wild-type *O. sativa* plants.

To rule out that this higher *Os*NifB^*Mt*^ activity was affecting cell growth and development and precluding the generation of plants expressing *Os*NifB^*Mt*^ (Figure 1), we generated more lines expressing *Os*NifB^*Mt*^. The MtB15 line expressed *Os*NifB^*Mt*^ at high levels (in addition to *Os*FdxN^*Av*^) not only in callus (Figures 4a and S6a) but also in leaves of the corresponding regenerated plants (Figures 4b and S6b), which indicates that expression of NifB^*Mt*^ is likely not detrimental to the plants. Similarly, NifB^*Mi*^ expression was shown to be stable in T1 plants of the MiB115 line (Figures 5 and S7).

## Discussion

The engineering of staple crops to fix nitrogen has been an important goal of plant biotechnology for several decades. If successful, this approach offers the potential to reduce or even abolish our dependence on nitrogen fertilizers, while maintaining the nitrogen content of soils. Natural BNF occurs only in some prokaryotes and is catalyzed by a nitrogenase complex with assistance from various accessory proteins required to assemble and incorporate metal cofactors into nitrogenase.^10^ Many of these components are extremely sensitive to O_2_, which is an additional challenge when transferring the trait to plants.^42^ One solution is to express the nitrogenase and its accessory proteins in the plant mitochondria, a strategy that would reduce O_2_ exposure and supply energy for nitrogenase activity and a ready source of [Fe–S] clusters generated by proteins similar to the bacterial NifUS system.^7^

Although many *nif* genes are involved in the assembly and activity of nitrogenase and its metal cofactors in bacteria, not all are expected to be required to reconstitute nitrogenase activity in plants because some of the accessory functions can be fulfilled by endogenous proteins.^43^ The minimal gene set that must be transferred to plants includes NifD, NifK, and NifH which form the nitrogenase enzyme and NifE, NifN, and NifB which catalyze essential reactions in the biosynthesis of FeMo-co, the active-site cofactor of nitrogenase.^10^ These six genes have been expressed in the mitochondria of *S. cerevisiae* and in some cases, also transiently in *N. benthamiana*(34,40,44−48) but have yet to be expressed in any staple crop.

The solubility of NifB is a prerequisite for its activity. Previous work on *S. cerevisiae* showed that the NifB proteins from both *M. thermautotrophicus* and *M. infernus* were soluble in the mitochondria and accumulated at high levels.^34^ We therefore generated rice plants co-expressing *Os*NifB^*Mi*^ or *Os*NifB^*Mt*^ with *Os*FdxN^*Av*^. In our initial experiments, we observed that the expression frequency and levels of *Os*NifB appeared to depend on the NifB variant. While we could detect *Os*NifB^*Mi*^ in three out of four callus lines we analyzed, *Os*NifB^*Mt*^ was only detectable in one out of four lines. At the plant level, protein accumulation could only be measured in *Os*NifB^*Mi*^ lines. We hypothesized that *Os*NifB^*Mt*^ might be detrimental to cell growth and development, thus limiting the number of plants able to regenerate, when expressing the protein. We therefore initiated new transformation experiments aiming to generate more lines expressing *Os*NifB^*Mt*^. Indeed, it proved to be difficult to generate additional lines expressing this protein. It is possible that NifB activity might interfere with other essential developmental processes in the cells or compete for essential precursors in metabolic processes sharing common precursors. However, we were able to obtain one line that accumulated *Os*NifB^*Mt*^ in both callus and regenerated plants (Figure 4). While moving forward with the nitrogenase engineering process, it would be desirable to circumvent this problem, for example, by using tissue-specific or regulated promoters.

Likely reasons to explain the different outcomes when expressing NifB^*Mi*^ and NifB^*Mt*^ in rice plants are not clear at present. An overlay of NifB^*Mi*^ and NifB^*Mt*^ structures (Figure S8) shows that secondary structure elements and relevant residues in both structures match, with only two differences: (1) the NifB^*Mi*^ H^22^ residue, which appears to stabilize the K-cluster,^21^ has been modeled by AlphaFold in the rotated position compared to its equivalent H^24^ residue in NifB^*Mt*^ and (2) the C-terminal stretch of NifB proteins. This region was well resolved in the NifB^*Mt*^ structure containing the K-cluster, but it was shown to be disordered before K-cluster formation (in the absence of K2-cluster) in the crystal structure of another Archaeal NifB homolog.^19^ It was proposed that the short C-terminal stretch acted as a strap closing the side of the NifB β-barrel structure and stabilizing the K2-cluster.^21^ It should also be noted that the confidence of the AlphaFold model for this region of NifB^*Mi*^ was low.

Earlier studies involved the co-expression of NifB with NifU, NifS, and FdxN in *S. cerevisiae*.^34,40^ Analysis of the *Sc*NifB^*Mi*^ protein isolated from different *S. cerevisiae* strains showed that while NifUS was important for providing [4Fe–4S] clusters, FdxN was more important for NifB activity.^34^ Earlier studies had shown that FdxN was required for efficient NifB-co biosynthesis,^23^ but its exact role is still unknown. Several non-exclusive roles in NifB Fe–S cluster acquisition or maturation or as a participant of the NifB reaction have been proposed.^10^ For example, NifB produced recombinantly in *S. cerevisiae* required FdxN to acquire the EPR signatures of its three clusters.^34^ FdxN could also promote the reductive coupling of K1- and K2-clusters to form the [8Fe–8S] K-cluster, a reaction intermediate of NifB-co synthesis.^21^ Finally, FdxN could serve as an electron donor to the NifB RS-cluster for the reductive cleavage of SAM and release of the dA• radical.

In contrast to NifB, NifH showed similar activity (400 nmol of C_2_H_4_ min^–1^ mg^–1^ NifDK) when co-expressed with either NifM alone or with NifM, NifS, and NifU in *S. cerevisiae*.^49^ This may reflect the distinct mechanisms used to incorporate [4Fe–4S] clusters into the NifB and NifH proteins or different requirements for these clusters. While NifH contains a permanent [4Fe–4S] cluster only required for catalysis, NifB requires a [4Fe–4S] cluster for catalysis and two additional [4Fe–4S] clusters as substrates for NifB-co biosynthesis.

The host organism can also influence the activity of Nif proteins. For example, when NifH was co-expressed with NifM, NifU, and NifS, it was functional in *S. cerevisiae* but not in*N. benthamiana*, and in the latter case, reconstitution in vitro was necessary to restore activity.^47^ Although NifU is the major provider of [4Fe–4S] clusters for nitrogenase in vivo, the*Klebsiella pneumoniaenifUS* double mutant still synthesizes NifB-co, albeit at lower levels compared to the wild-type stain.^18^ This shows that [4Fe–4S] clusters for NifB-co biosynthesis can be provided by other sources, such as the iron–sulfur cluster assembly or sulfur mobilization systems.^18,50^ The IscS protein purified from an *A. vinelandii* strain with deleted *nifS* catalyzes the same reaction as NifS.^51^ We have been unable to obtain detectable protein accumulation of NifU^*Av*^ and NifS^*Av*^ in transformed rice, and this study was therefore limited to the expression of NifB and FdxN. The identification of NifU and NifS variants suitable for expression in rice should therefore be the focus of future studies.

In conclusion, we were able to express the *M. infernus* NifB, *M. thermautotrophicus* NifB, and *A. vinelandii* FdxN proteins in rice using the Cox4 peptide to ensure the efficient targeting of all three proteins to the mitochondria, where they were correctly processed. The enzymatic activity of purified *Os*NifB proteins was confirmed by using the in vitro FeMo-co synthesis assay. We therefore show that these two NifB proteins fulfil the requirements for functional NifB in the rice mitochondria, such as stability, solubility, and competence, to acquire [4Fe–4S] cluster substrates, but further research is required to demonstrate active NifB *in planta*. This may require the co-expression of additional *nif* genes. Nevertheless, the expression of functional NifB in this study is an important step toward the engineering of nitrogenase activity in cereals.

## Materials and Methods

### Construct Preparation

The sequences of *M. infernusnifB* (*nifB*^*Mi*^), *M. thermautotrophicusnifB* (*nifB*^*Mt*^), and *A. vinelandiifdxN* (*fdxN*^*Av*^), the mitochondrial targeting peptide Cox4, and the TS and HA tags were codon-optimized for *S. cerevisiae* using the GeneOptimizer tool (Thermo Fisher Scientific, Waltham, MA, USA) and synthesized by Thermo Fisher Scientific, as described.^34,40^ The empty vector pUC57 (GenScript Biotech, Piscataway, NJ, USA) was digested with *Acc*65I and *Sal*I, allowing the insertion of the *ZmUbi1* + *1st i* promoter. The *Cox4-TS*-*nifB*^*Mi*^-nos cassette was generated by PCR using pN2XJ21^40^ as the template and was introduced into the intermediate vector pUC57-*ZmUbi1* + *1st i* at the *Sal*I and *Sph*I sites to produce pMiNifB. The pMiNifB vector was digested with *BamH*I and *BstE*II, allowing the insertion of the *nifB*^*Mt*^ sequence generated by PCR using pN2SB103^34^ as the template, to produce pMtNifB. The pMiNifB vector was digested with *Sal*I and *BstE*II, allowing insertion of the synthetic *Cox4-fdxN*^*Av*^*-HA* cassette, to generate pAvFdxN. All restriction enzymes and T4 DNA ligase were obtained from Promega (Madison, WI, USA) or New England Biolabs (Ipswich, MA, USA). The plasmids were amplified in *E. coli* DH5α cells grown at 37 °C in lysogeny broth medium supplemented with 100 μg/mL ampicillin. The fidelity of DNA constructs was verified by Sanger sequencing (Stabvida, Caparica, Portugal). The sequences of cloning primers and DNA constructs for rice expression are listed in Tables S1 and S2.

### Transformation of Rice Explants, Callus Recovery, and Regeneration of Transgenic Plants

Seven-day-old mature rice embryos (*Oryza sativa* cv. Nipponbare) were isolated as explants for particle bombardment. The embryos were transferred to Murashige & Skoog (MS) osmoticum (MSO) medium for 4 h in the dark before transformation with 10 mg gold particles coated with the transgene constructs (pMiNifB and pAvFdxN or pMtNifB and pAvFdxN) and the hygromycin phosphotransferase (*hpt*) selectable marker at a molar ratio of 3:3:1. The bombarded embryos were maintained on MSO medium for 16 h in the dark and then transferred to MS selection medium for 4 weeks in the dark, with one subculture after 2 weeks. Half of the resistant callus was kept under selection, and the other half was transferred to MS regeneration medium with a 12 h photoperiod for 3–4 weeks to regenerate transgenic plantlets. The transgenic plantlets were transferred to rooting medium (HMS) with a 12 h photoperiod for 2 weeks and planted to soil in the greenhouse with a 12 h photoperiod and 80% relative humidity. Media compositions are listed in Table S3.

### Protein Extraction and Immunoblot Analysis

Soluble rice leaf protein extracts were prepared by grinding ca. 50 mg rice tissue (snap-frozen in liquid N_2_) in 2 mL Eppendorf tubes using 3 mm BeadBug steel balls and a microtube homogenizer (Benchmark Scientific, Edison, NJ, USA) operating at 400 rpm for 20 s. Leaf powder was resuspended in 7 volumes (v/w) of extraction buffer comprising 100 mM Tris-HCl (pH 8.6), 200 mM NaCl, 10% glycerol, 1 mM PMSF, 1 μg/mL leupeptin, and 5 mM EDTA and homogenized twice. Cell debris was removed by centrifugation (20,000*g*, 5 min, 4 °C), and the supernatant was collected and stored at −80 °C. Soluble rice callus extracts were prepared using a blender (see STAC purification section).

Rice proteins were separated by SDS-PAGE and then immunoblotted to Protran Premium 0.45 μm nitrocellulose membranes (GE Healthcare, Chicago, IL, USA) using a semi-dry transfer apparatus (Bio-Rad Laboratories, Hercules, CA, USA) at 20 V for 45 min. Loading equivalence was confirmed by staining polyacrylamide gels with Coomassie brilliant blue or nitrocellulose membranes with Ponceau S. The membranes were blocked with 5% non-fat milk in 20 mM Tris-HCl (pH 7.5), 150 mM NaCl, and 0.02% Tween-20 (TBS-T) for 1 h at room temperature, before incubation with primary antibodies overnight at 4 °C. Primary polyclonal antibodies against NifB^*Mi*^ (generated in-house), and monoclonal antibodies against the strep-tag II (2-1507-001, IBA Lifesciences, Göttingen, Germany) and the HA tag (H6908, Sigma-Aldrich, St Louis, MO, USA) or Rubisco (AS03 037A, Agrisera, Vännäs, Sweden, used as loading control) were diluted at 1:2,000–1:5,000 in TBS-T supplemented with 5% bovine serum albumin (BSA). Secondary antibodies (Thermo Fisher Scientific) were diluted at 1:20,000 in TBS-T supplemented with 2% non-fat milk and incubated for 2 h at room temperature. Membranes were developed on medical X-ray films (AGFA, Mortsel, Belgium) using enhanced chemiluminescence.

### Purification of *Os*NifB^*Mi*^ and *Os*NifB^*Mt*^ by Strep-Tag Affinity Chromatography

*Os*NifB^*Mi*^ and *Os*NifB^*Mt*^ were prepared for STAC purification at O_2_ levels below 1 ppm in an anaerobic chamber (Coy Laboratory Products, Grass Lake, MI, USA or MBraun, Garching, Germany). Callus was disrupted in lysis buffer comprising 100 mM Tris-HCl (pH 8.5), 300 mM NaCl, 10% glycerol, 3 mM sodium dithionite (DTH), 5 mM 2-mercaptoethanol, 1 mM PMSF, 1 μg/mL leupeptin, 10 μg/mL DNAse I, and 1:200 (v/v) BioLock solution (IBA Lifesciences Göttingen, Germany) at a ratio of 1:3 (w/v). Total extracts were prepared by lysing the cell suspensions under anaerobic conditions using the Oster 4655 blender (Newell Brands, Atlanta, GA, USA) modified with a water-cooling system operating at full speed in 4 cycles of 2 min at 4 °C. Extracts were transferred to centrifuge tubes equipped with sealing closures (Beckman Coulter, Brea, CA, USA) and centrifuged (50,000*g*, 1.5 h, 4 °C) using the Beckman Coulter Avanti J-26 XP device. The supernatant was passed through Nalgene 0.2 μm filter cups (Thermo Fisher Scientific) to yield a cell-free extract of soluble proteins. This was loaded at 2.5 mL/min onto a 5 mL Strep-Tactin XP column (IBA Lifesciences) attached to an ÄKTA FPLC system (GE Healthcare). The column was washed with 150 mL of 100 mM Tris-HCl (pH 8.0), 300 mM NaCl, 10% glycerol, 2 mM DTH, and 5 mM 2-mercaptoethanol at 16 °C, and bound proteins were eluted with 15–20 mL of the same wash buffer supplemented with 50 mM biotin (IBA LifeSciences). The elution fraction was concentrated using the Amicon Ultra centrifugal filter (Millipore Sigma, Burlington, MA, USA) with a cut-off size of 10 kDa. Biotin was removed by passing the protein through PD-10 desalting columns (GE Healthcare) equilibrated with wash buffer. The desalted eluate was concentrated and snap-frozen in Nalgene cryovials and stored in liquid nitrogen.

### Quantification of Purified *Os*NifB^*Mi*^ and *Os*NifB^*Mt*^ Proteins and N-Terminal Sequencing

The yield of purified *Os*NifB^*Mi*^ and *Os*NifB^*Mt*^ was determined by Coomassie gel titration against standards of the purified *S. cerevisiae* NifB (*Sc*NifB^*Mi*^ and *Sc*NifB^*Mt*^) protein, as shown in Figure S5. Amino terminal amino acid sequencing was performed by Edman degradation (Centro de Investigaciones Biológicas, Madrid, Spain). 25 pmol *Os*NifB protein was separated by SDS-PAGE, transferred to 0.2 μm Sequi-Blot PVDF membranes (Thermo Fisher Scientific) in 50 mM borate buffer (pH 9.0), stained with freshly prepared 0.1% Coomassie R-250 (Sigma-Aldrich) in 40% methanol and 10% acetic acid, and then destained using 50% methanol.

### FeMo-Co Synthesis and Apo-NifDK Reconstitution In Vitro

FeMo-co synthesis and apo-NifDK reconstitution assays were carried out in vitro in an anaerobic chamber, as previously described.^34^ For the in vitro synthesis of FeMo-co, each 100 μL reaction contained 3 μM NifH^*Av*^, 1 μM *Os*NifB, 1.5 μM apo-NifEN^*Av*^, 0.6 μM apo-NifDK^*Av*^, 17.5 μM Na_2_MoO_4_, 175 μM *R*-homocitrate, 9 μM [Fe_4_–S_4_]cluster-loaded NifU^*Ec*^ (holo-NifU^*Ec*^), 125 μM SAM, 1 mg/mL BSA, and the ATP-regenerating mixture (1.23 mM ATP, 18 mM phosphocreatine disodium salt, 2.2 mM MgCl_2_, 3 mM DTH, 46 μg/mL creatine phosphokinase). For the positive control FeMo-co synthesis assay, holo-NifU^*Ec*^ was omitted, and *Os*NifB was replaced with 2.5 μM NifB-co. The reactants were incubated for 60 min at 30 °C. For the acetylene reduction assays, 500 μL of the ATP-regenerating mixture and 2.0 μM NifH^*Av*^ were added to the reaction tube. The reaction mixture was then transferred to 9 mL serum vials under an argon/acetylene (94%/6%) atmosphere. The reaction was incubated for 20 min at 30 °C. To measure ethylene formation, 50 μL of the gas phase was taken from the reaction vials and injected in the Shimadzu GC-2014 gas chromatographer equipped with the Porapak N 80/100 column (Shimadzu, Kyoto, Japan).

### Statistical Analysis

Standard deviation (SD) of in vitro activity data was calculated based on two biological replicates (each one with two technical replicates).

## Supporting Information Available

Sequences of primers, vectors, and constructs used in this study, composition of media for in vitro culture, levels of *nifB* and *fdxN* transcripts in individual transgenic rice lines, quantification of purified rice NifB proteins, uncropped immunoblots and Ponceau-stained membranes, and overlay of NifB^*Mi*^ and NifB^*Mt*^ protein structures (PDF)

## Notes

Data Availability Statement: the authors declare that the data supporting the findings of this study are available within the article, its Supporting Information and data, and upon request.
