# Supplementary material for: Nitrogenase Cofactor Maturase NifB Isolated from Transgenic Rice is Active in FeMo-co Synthesis
Source: ACS Synth Biol. 2022 Aug 23;11(9):3028–36. doi: 10.1021/acssynbio.2c00194 (PMC9486962; doi:10.1021/acssynbio.2c00194)
Supplement: Supplementary file 1 — sb2c00194_si_001.pdf [file sb2c00194_si_001.pdf]

Supplementary Information to

Nitrogenase Cofactor Maturase NifB Isolated from Transgenic Rice is Active in FeMo-co Synthesis

Wenshu He[1≠], Stefan Burén[2,3≠], Can Baysal[1≠], Xi Jiang[2,3], Teresa Capell[1], Paul Christou[1,4]* and Luis M. Rubio[2,3]*

[1]Department of Plant Production and Forestry Science, University of Lleida-Agrotecnio CERCA Center, 25198 Lleida, Spain

[2]Centro de Biotecnología y Genómica de Plantas, Universidad Politécnica de Madrid (UPM), Instituto Nacional de Investigación y Tecnología Agraria y Alimentaria (INIA), 28223 Madrid, Spain

[3]Departamento de Biotecnología-Biología Vegetal, Escuela Técnica Superior de Ingeniería Agronómica, Alimentaria y de Biosistemas, Universidad Politécnica de Madrid, 28040 Madrid, Spain

[4]ICREA, Catalan Institute for Research and Advanced Studies, 08010 Barcelona, Spain

*Luis M. Rubio. Email: lm.rubio@upm.es

*Paul Christou. Email: paul.christou@udl.cat

[≠]Authors contributed equally to this work

Contents:

Figure S1. Quantification of *nifB* and *fdxN* mRNA in representative *Os*NifB^{Mi} and *Os*NifB^{Mt} lines.

Figure S2. Uncropped immunoblots and Ponceau stained membranes shown in Figure 1a.

Figure S3. Uncropped immunoblots and Ponceau stained membranes shown in Figure 1b.

Figure S4. Uncropped immunoblots and Ponceau stained membranes shown in Figures 2a-c.

Figure S5. Coomassie staining and quantification of *Os*NifB^{Mi} and *Os*NifB^{Mt} purified from line MiB32, line MiB115, and line MtB35.

Figure S6. Uncropped immunoblots and Ponceau stained membranes shown in Figure 4.

Figure S7. Uncropped immunoblots and Ponceau stained membranes shown in Figure 5.

Figure S8. Overlay of NifB^{Mi} and NifB^{Mt} structures shown as ribbon diagrams.

Table S1. List of vectors and constructs used in this study.

Table S2. Primers used for vector construction.

Table S3. Composition of media for *in vitro* culture.

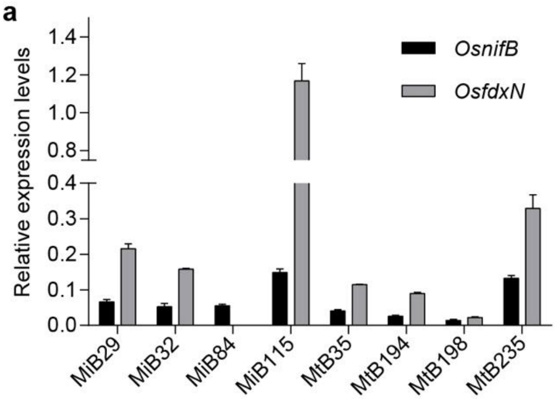

b

Gene	Primer sequence (5′→3′)
actin^{Os}	F: TCATGTCCCTCACAATTTCC<br>R: GACTCTGGTGATGGTGTCAGC
nifB^{Mi}	F: CTGTCAACGCCATTAAGCCA<br>R: TCGGAGGCCTTCTTGATACCT
nifB^{Mt}	F: ACGGTTTGTTGTTGCCAGAT<br>R: GCCAACTTTTCAATGCCTTCC
fdxN^{Av}	F: GCTGGGCATGTGTTGATGTG<br>R: GCAGGAGTTCCGTCTGCTAA

Figure S1. Quantification of *nifB* and *fdxN* mRNA in representative *Os*NifB^{Mi} and *Os*NifB^{Mt} lines. Four independent lines from each transgene were used for quantitative real-time PCR (qRT-PCR) analysis**.** (a) Relative mRNA expression levels in rice callus (normalized to *OsActin*). Data are means ± SD (n = 3). (b) Primers used for qRT-PCR analysis. Abbreviations: *OsnifB*: *O. sativa*-derived *M. infernus nifB* (*nifB*^{Mi}) and *O. sativa*-derived *M. thermautotrophicus nifB* (*nifB*^{Mt}); *OsfdxN*: *O. sativa*-derived *A. vinelandii fdxN* (*fdxN*^{Av}). MiB29, MiB32, MiB84, MiB115, MtB35, MtB194, MtB198, and MtB235 are eight independent lines.

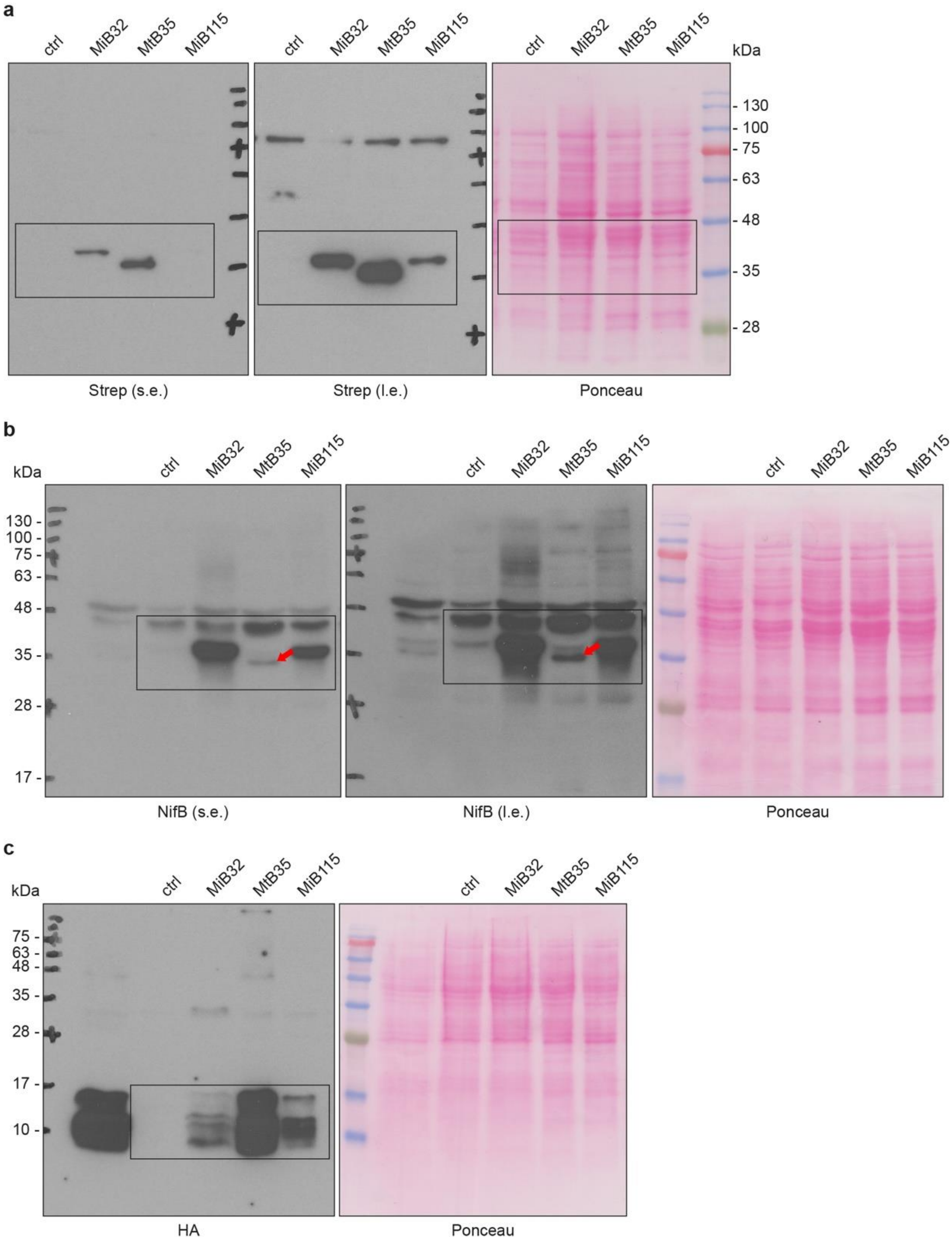

Figure S2. Uncropped immunoblots and Ponceau stained membranes shown in Figure 1a. Membranes probed with antibodies against the TS tag (a), NifB (b), and HA (c). *Os*NifB^{Mi} and *Os*NifB^{Mt} were detected with antibodies against NifB^{Mi} and the N-terminal TS tag. *Os*FdxN^{Av} was detected with antibodies against the C-terminal HA tag. The red arrow indicates the signal from *Os*NifB^{Mt}. Abbreviations: s.e.: short exposure during immunoblot detection; l.e.: long exposure during immunoblot detection; MiB32, MiB115, and MtB35 are three independent lines.

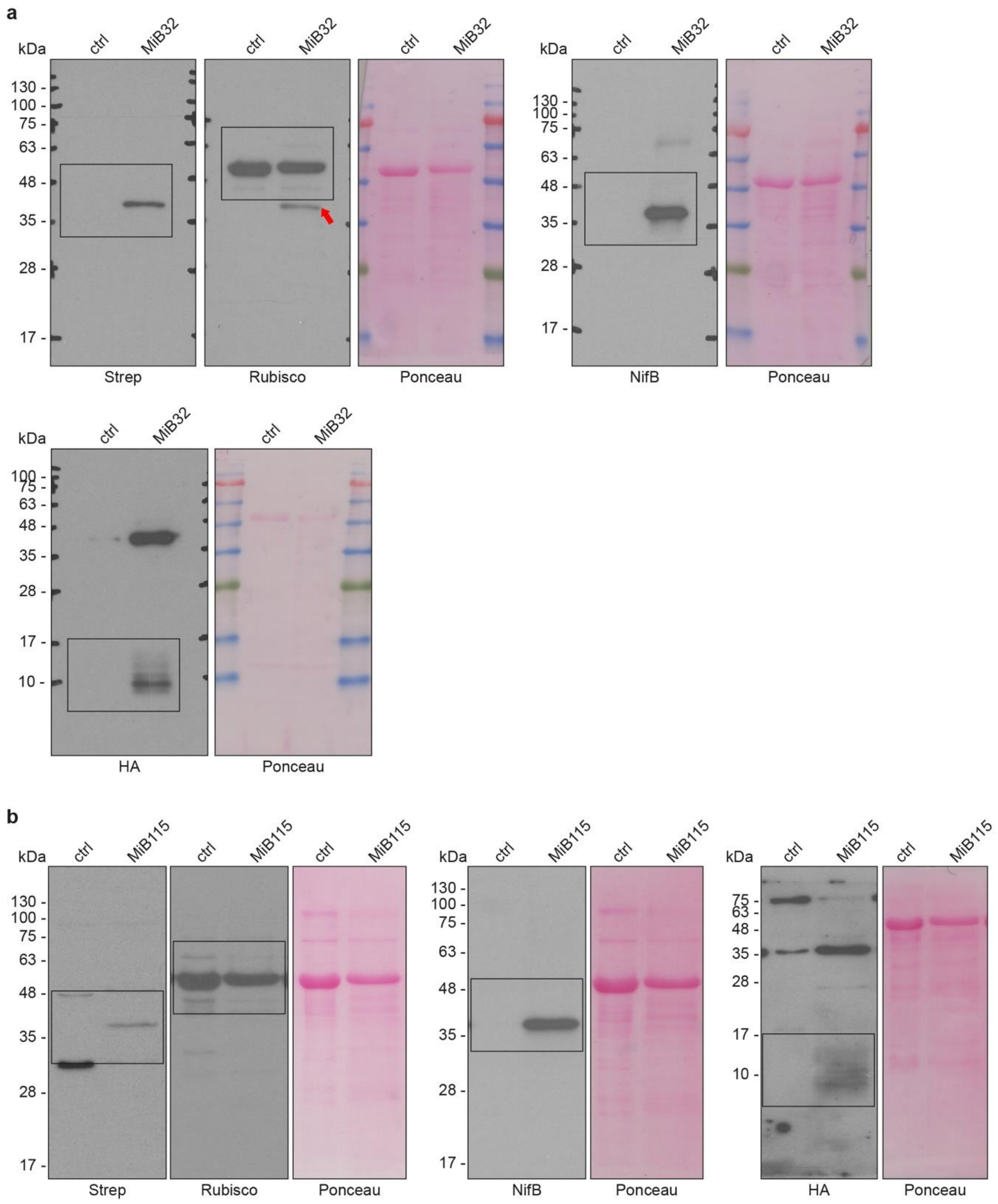

Figure S3. Uncropped immunoblots and Ponceau stained membranes shown in Figure 1b. (a) Line MiB32 and (b) line MiB115. *Os*NifB^{Mi} was detected with antibodies against NifB^{Mi} and the N-terminal TS tag. *Os*FdxN^{Av} was detected with antibodies against the C-terminal HA tag. The red arrow indicates the signal originating from *Os*NifB^{Mi} detected using the Strep antibodies upon reprobing the membrane with antibodies against Rubisco. Abbreviations: MiB32 and MiB115 are two independent lines.

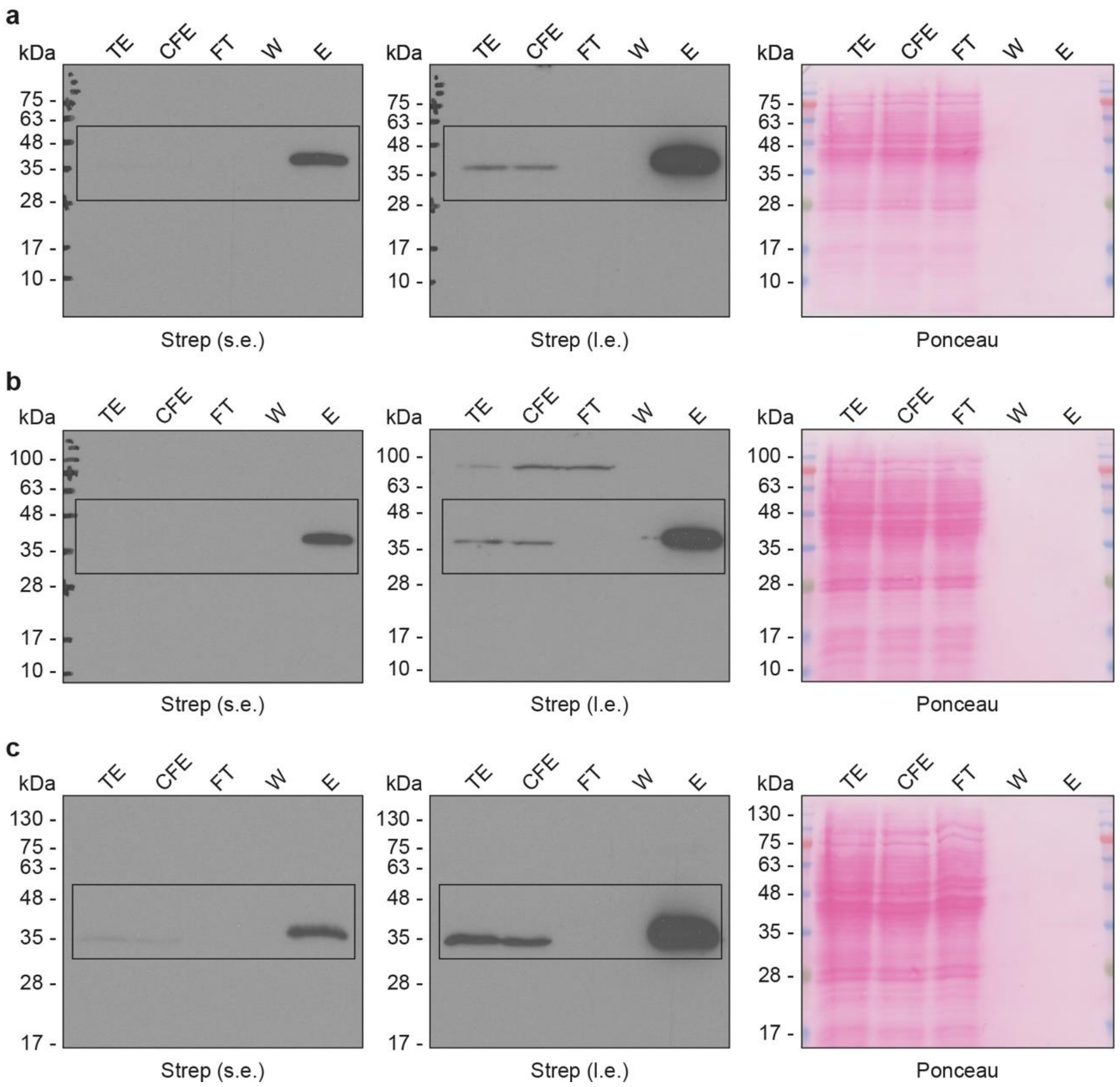

Figure S4. Uncropped immunoblots and Ponceau stained membranes shown in Figures 2a (a), 2b (b), and 2c (c). STAC purification fractions from line MiB32 (a), line MiB115 (b), and line MtB35 (c). *Os*NifB^{Mi} and *Os*NifB^{Mt} were detected with antibodies against the N-terminal TS tag. Abbreviations: TE: total extract, CFE: soluble cell-free extract, FT: flow-through fraction, W: wash fraction, E: elution fraction. s.e.: short exposure during immunoblots detection; l.e.: long exposure during immunoblot detection; MiB32, MiB115, and MtB35 are three independent lines.

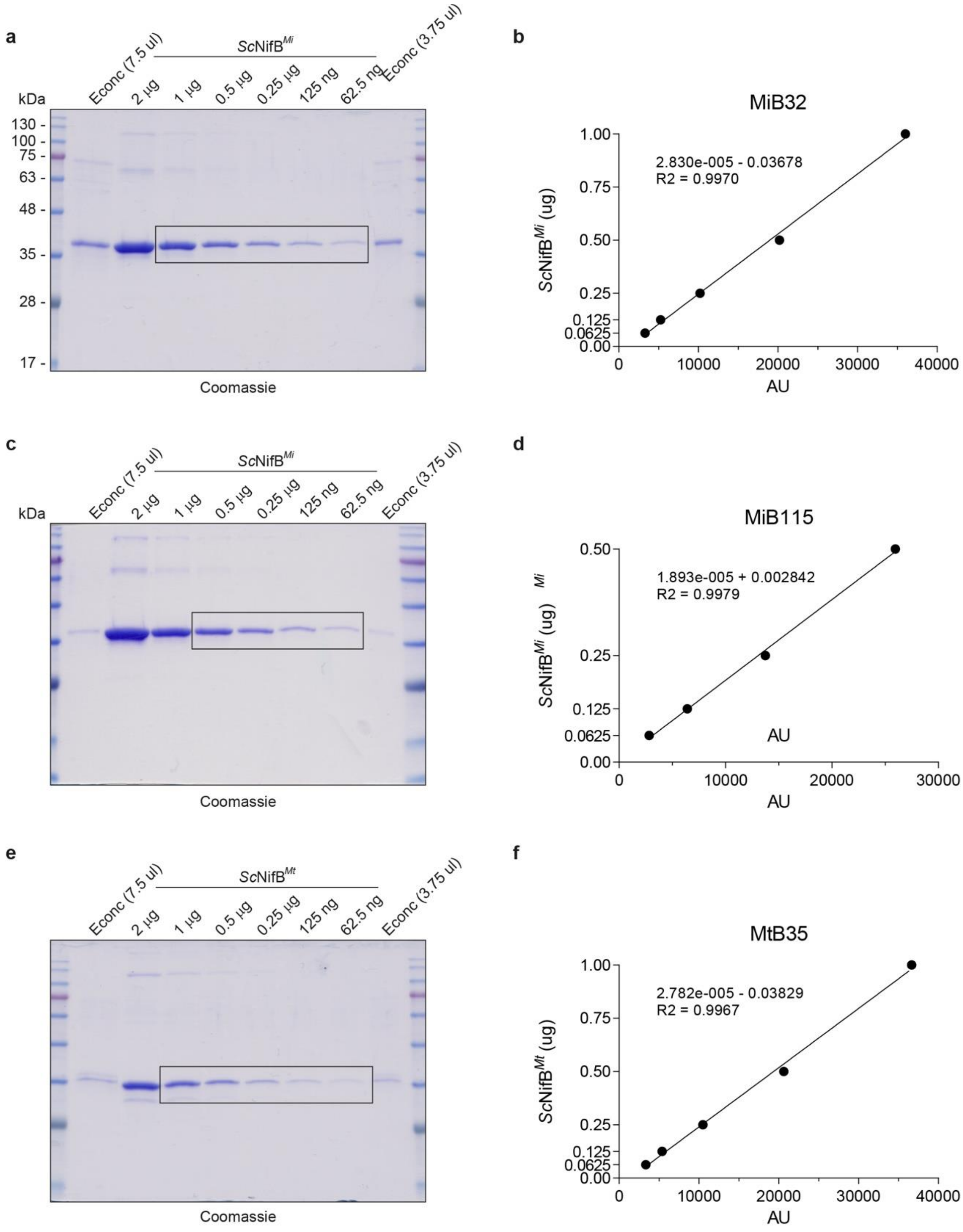

Figure S5. Coomassie staining and quantification of *Os*NifB^{Mi} and *Os*NifB^{Mt} purified from line MiB32 (a and b), line MiB115 (c and d), and line MtB35 (e and f). Coomassie stained gels were loaded with known amounts of *Sc*NifB^{Mi} (a and c) and *Sc*NifB^{Mt} (e) to quantify the concentration of the eluted and concentrated *Os*NifB^{Mi} (b and d) and *Os*NifB^{Mt} (f) using linear regression. The slope, intercept and the R^2 for the linear regression analyses are indicated. Black boxes indicate the Coomassie stained *Sc*NifB proteins used for the analyses.

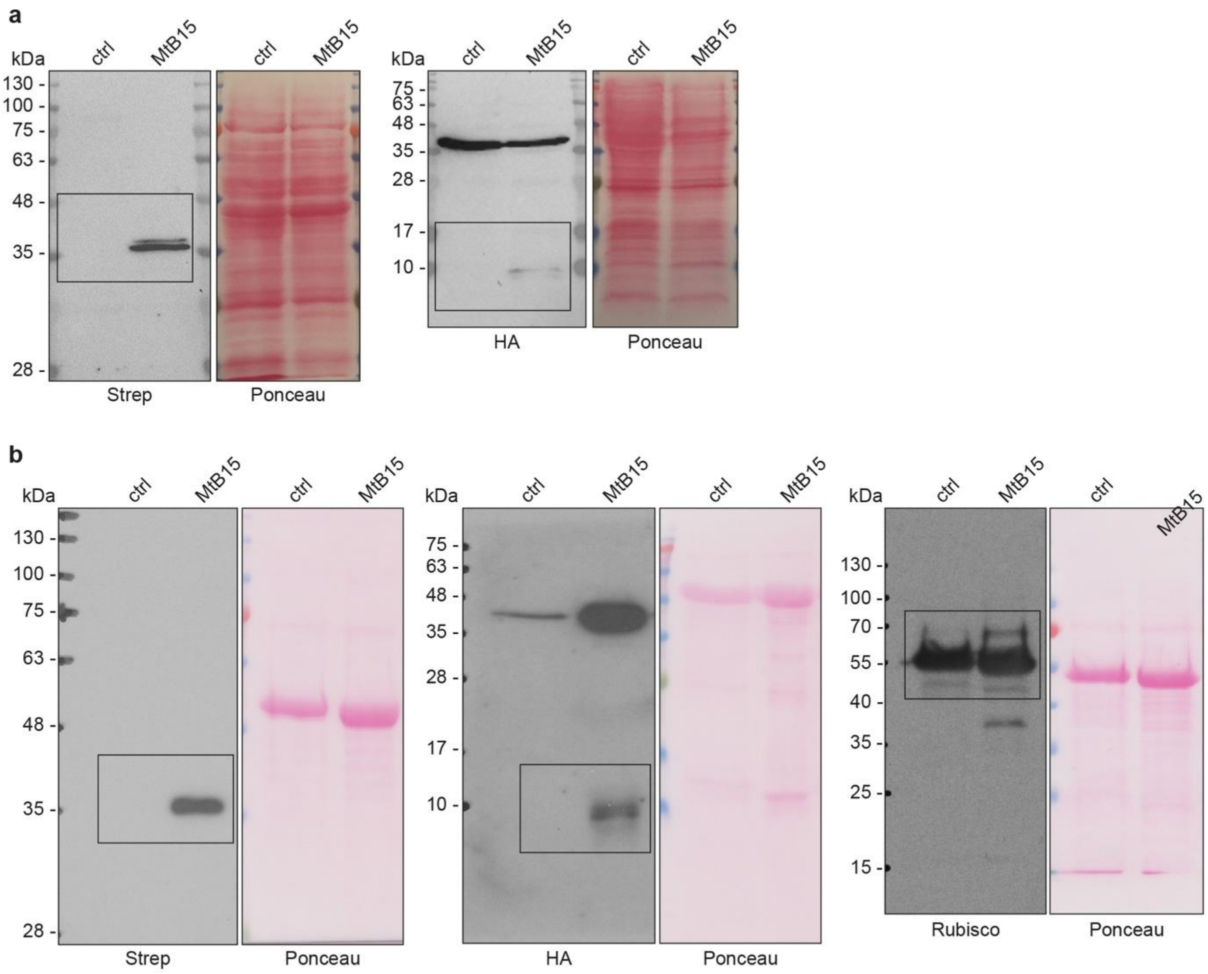

Figure S6. Uncropped immunoblots and Ponceau stained membranes shown in Figure 4. Line MtB15 callus (a) and plant (b). *Os*NifB^{Mt} was detected with antibodies against the N-terminal TS tag. *Os*FdxN^{Av} was detected with antibodies against the C-terminal HA tag. Abbreviations: MtB15 is a line accumulating NifB^{Mt} in callus and regenerated plants.

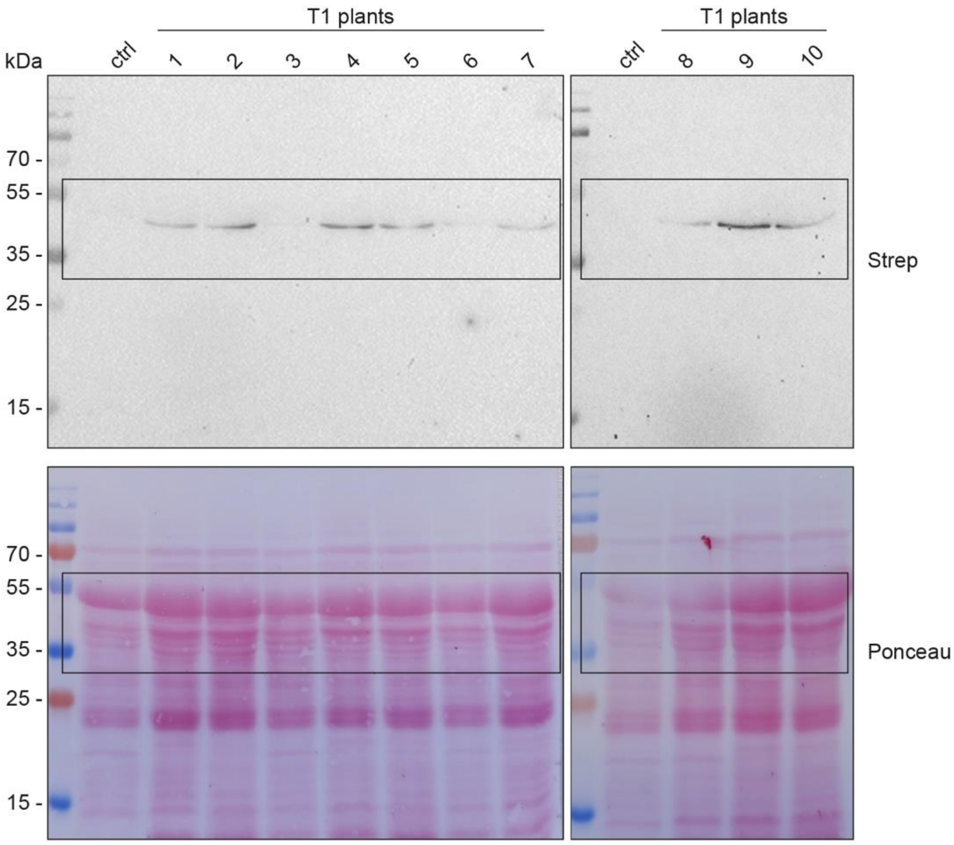

Figure S7. Uncropped immunoblots and Ponceau stained membranes shown in Figure 5. Soluble leaf extracts from 10 different T1 plants of the MiB115 line accumulating *Os*NifB^{Mi} were probed with antibodies against the N-terminal TS tag. The control lane was loaded with soluble leaf extract obtained from wild-type *O. sativa* plants.

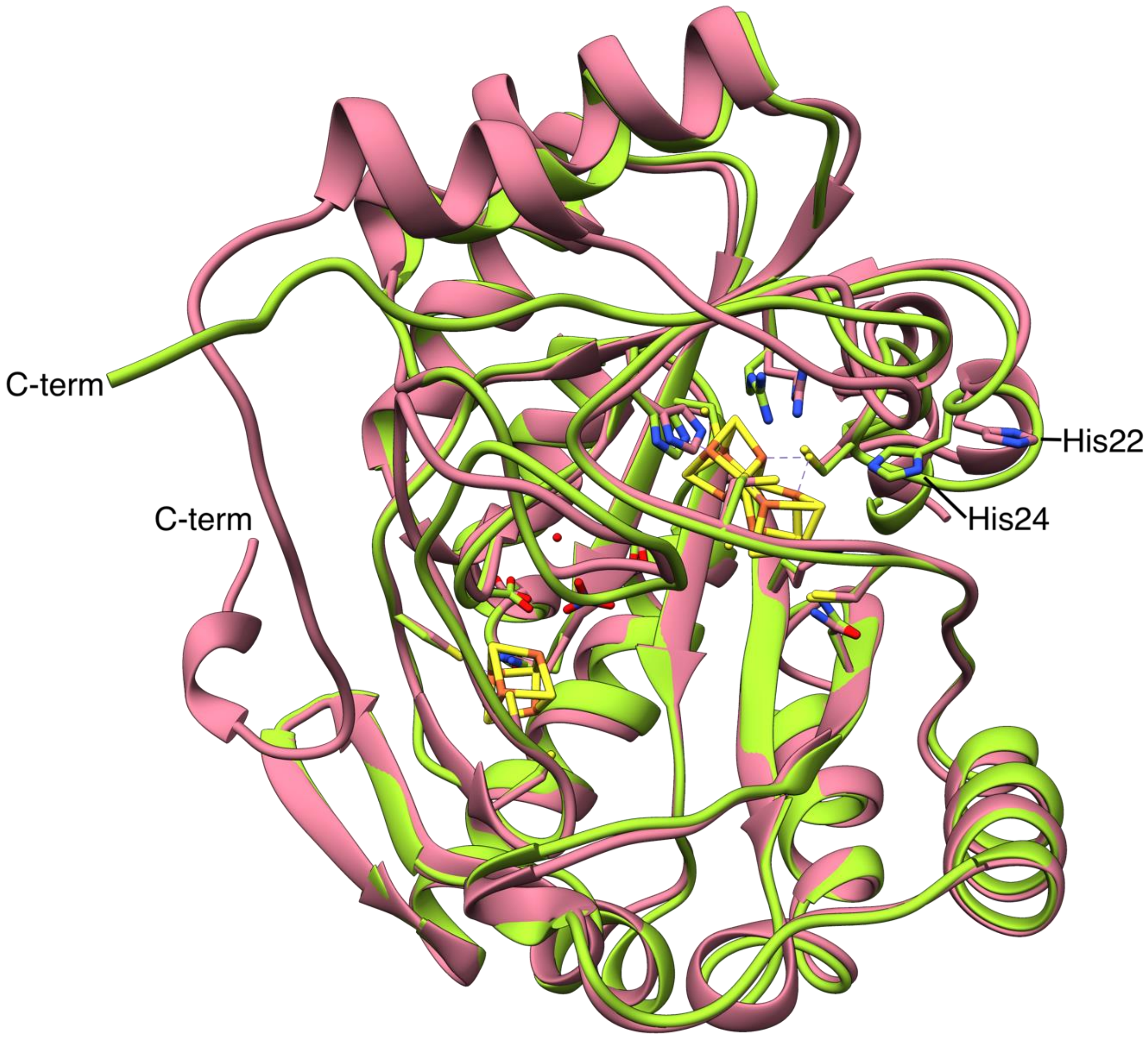

Figure S8. Overlay of NifB^{Mi} and NifB^{Mt} structures shown as ribbon diagrams. The NifB^{Mt} structure (green) was solved by X-ray crystallography.[1] The NifB^{Mi} structure (magenta) is an AlphaFold prediction of UniProt D5VRM1. The RS cluster and the [8Fe-8S] K-cluster, formed by the fusion of K1- and K2-clusters, are shown in sticks together with residues important for their coordination or stability.[1]

References

(1) Jenner, L. P., Cherrier, M. V., Amara, P., Rubio, L. M., and Nicolet, Y. (2021) An unexpected P-cluster like intermediate en route to the nitrogenase FeMo-co, *Chem. Sci*. 12, 5269-5274.

Table S1. List of vectors and constructs used in this study. (a) Plant expression vectors. (b) Sequences of genetic constructs.

a) Plant expression vectors

Plasmids	Expressed proteins	Promoter / terminator	Size (kDa, with tag)
PUC57	Cox4-TS-NifB^{Mi}	p*ZmUbi1+1*st*i* / tNos	38
PUC57	Cox4-TS-NifB^{Mt}	p*ZmUbi1+1*st*i* / tNos	35
PUC57	Cox4-TS-FdxN^{Av}	p*ZmUbi1+1*st*i* / tNos	10

b) Sequences of genetic constructs

Cox4:
ATGCTTTCACTTAGACAATCTATTAGATTTTTCAAGCCAGCTACAAGAACTTTG
TGTTCTTCTAGATATCTTCTTCAGCAAAAACCT
MLSLRQSIRFFKPATRTLCSSRYLLQQKP

TS:
TGGAGTCATCCTCAGTTTGAGAAAGGTGGAGGTTCAGGTGGTGGAAGCGGTGG
ATCTGCTTGGTCACATCCACAATTTGAAAAA
WSHPQFEKGGGSGGGSGGSAWSHPQFEK

HA:
TATCCATATGATGTTCCAGATTATGCTTAA
YPYDVPDYA

NifB^{Mi}:
ATGGAAAAGATGTCCAAGTTCTCCCATTTGTTGAAAGCTCATCCATGCTTCAAC
GAAAAGGTTCATGATAAGTACGGTAGAGTTCATTTGCCAGTTGCTCCAAGATGT
AACATTGCTTGTAAGTTCTGCAAGAGGTCCGTTTCTAAAGAATGTTGTGAACAT
AGACCAGGTGTTTCTTTGGGTGTTTTGAAACCAGAAGATGTTGAGGACTACCTG
AAAAAGATCTTGAAAGAGATGCCAAACATCAAGGTTGTTGGTATTGCTGGTCC
TGGTGATTCTCTGTTTAACAAAGAAACTTTCGAAACCCTGAAGATCATCGACGA
AAAGTTTCCCAACTTGATTAAGTGCATTTCCACCAACGGTCTGTTGTTGTCTAA
GTACTACAAGGATTTGGCCAACTTGAACGTTAGAACTATTACCGTTACTGTCAA
CGCCATTAAGCCAGAAATCTTGGAAAAAATCGTTGACTGGGTTTACTACGACA
AGAAGTTGTATAGAGGTTTGGAAGGTGCCAAGTTGTTGATCGAAAAACAAATC
GAAGGTATCAAGAAGGCCTCCGAAGAAGATTTCATTATCAAGATCAACACCGT
CTTGATCCCAGAAATCAACATGGATCACGTTGTTGAAATTGCCAAGTTCTTCAA
GGATTACGCCTACGTTCAAAACATCATTCCATTGATTCCACAGTACAAGATGAA
GGAATTGAGAGCACCAACTTGCGAAGAAATCAAAAAGGTCAGAAAAGAGTGC
GAGAAGTACATCCCACAATTCAGAGCTTGTGGTCAATGTAGAGCTGATGCTGTT

GGTCTGATCAAAGAAAAAGAGCTGTTGAAAGAGTTTTTCAAAGAGAAGAACAA<br>AGAAAAGAACATCAAGCTGGAAGTGTTCGACTTGAAGCACTTCTCTCAT<br>MEKMSKFSHLLKAHPCFNEKVHDKYGRVHLPVAPRCNIACKFCKRSVSKECCEHR<br>PGVSLGVLKPEDVEDYLKKILKEMPNIKVVGIAGPGDSLFNKETFETLKIIDEKFPNL<br>IKCISTNGLLLSKYYKDLANLNVRTITVTVNAIKPEILEKIVDWVYYDKKLYRGLEG<br>AKLLIEKQIEGIKKASEEDFIIKINTVLIPEINMDHVVEIAKFFKDYAYVQNIIPLIPQY<br>KMKELRAPTCEEIKKVRKECEKYIPQFRACGQCRADAVGLIKEKELLKEFFKEKNK<br>EKNIKLEVFDLKHFSH
NifB^{Mt}:<br>ATGCCAGATCAAAGACAAACCAGATTCGCTCATATTACTAAGGCTCATCCATGC<br>TTCAACGAAAAGTTGCATGATAGAGTTGGTAGAGTTCATGTTCCAATTGCTCCA<br>AGATGTAACATCCATTGCAAGTTCTGTACCAGAGATATCAACGAATGTGAAAG<br>ACGTCCAGGTGTTACTGGTAGATTGATGACTGCTGATGATGCTATTAAGCACGT<br>CGAAAAGGTCAAAGAAGAAATGCCAATTTCCGTTATTGGTGTTGCTGGTCCAG<br>GTGATGCTTTGGCTAATGAAGAAACTTTCGAGTTCTTCAAGAAGGCCTCTAAGA<br>AGTTTCCAGATTTGTTGAAGTGTATGTCCACCAACGGTTTGTTGTTGCCAGATA<br>GAGCTGATGAATTGGCTGAATTGGGTATTAACACTGTTACTGTTACCGTTAACG<br>CTGTTGATCCAGAAATTGGTGAAAAGATCTACTCCTTCGTTGTCTACAAGGATA<br>AGGTTTATCATGGTAGAGAAGCCTTCGAAGTGTTGTCTAGAAATCAATTGGAA<br>GGCATTGAAAAGTTGGCCGAAAGAGGTATTATCGTCAAGGTTAACTCTGTTTTG<br>ATCCCAGGTTTGAACGATGAACATATTGTCGATATTGCCCGTGAAGTTAAGAAA<br>AGGGGTGCTTCTTTGATGAACATCATTCCATTGATTCCAATGGGTGAGATGAAG<br>GATTATCCAAGACCAACCTGTGAACAAATCGAAAGAGTTAGAAACGAAGTCGA<br>GAAGATCATCCCAGTTTTTAGAGCTTGTACTCAATGTAGAGCAGATGCTTATGG<br>TATCCCAGGTAAAAAAGAAGCTGATAAGCACTTGGATATGACCCCAGCTTCTC<br>ATTACTAA<br>MPDQRQTRFAHITKAHPCFNEKLHDRVGRVHVPIAPRCNIHCKFCTRDINECERRP<br>GVTGRLMTADDAIKHVEKVKEEMPISVIGVAGPGDALANEETFEFFKKASKKFPDL<br>LKCMSTNGLLLPDRADELAELGINTVTVTVNAVDPEIGEKIYSFVVYKDKVYHGRE<br>AFEVLSRNQLEGIEKLAERGIIVKVNSVLIPGLNDEHIVDIAREVKKRGASLMNIIPLI<br>PMGEMKDYPRPTCEQIERVRNEVEKIIPVFRACTQCRADAYGIPGKKEADKHLDMT<br>PASHY*
FdxN^{Av}:<br>ATGGCTCTTAAGATAGTTGAGTCTTGTGTGAACTGCTGGGCATGTGTTGATGTG<br>TGCCCAAGTGAGGCTATATCCTTGGCAGGTCCTCATTTTGAAATTTCTGCTTCA<br>AAATGCACCGAGTGTGATGGAGACTATGCTGAAAAGCAATGCGCATCTATTTG<br>TCCAGTTGAAGGTGCTATCTTGTTAGCAGACGGAACTCCTGCTAACCCACCTGG<br>TTCACTTACAGGAATCCCACCTGAAAGATTGGCTGAGGCAATGAGAGAAATAC<br>AGGCAAGG<br>MALKIVESCVNCWACVDVCPSEAISLAGPHFEISASKCTECDGDYAEKQCASICPV<br>EGAILLADGTPANPPGSLTGIPPERLAEAMREIQAR

Table S2. Primers used for vector construction.

Primers used for vector construction		
Genetic element	Primer sequence (5′→3′)	Restriction enzymes
p*ZmUbi1+1*st*i*	F: TAAGCAGGATCCGGAGTGCAGTGCAGCGTGA<br>R: TGCTTACTGCAGAAGTAACACCAAACAACAG	*Acc*65I<br>*Sal*I
Cox4-TS-nifB^{Mi}*-nos*	F: TAAGCACTCGACATGCTTTCACTTAGACAATC<br>R: TGCTTAGCATGA GATCTAGTAACATAGATGAC	*Sal*I<br>*Sph*I
NifB^{Mt}	F: TAAGCAGGATCCATGCCAGATCAAAGACAAAC<br>R: TGCTTAGGTNACCTTAGTAATGAGAAGCTGGGG	*BamH*I<br>*BstE*11
Cox4-fdxN^{Av}*-HA*	F: TAAGCACTCGACATGCTTTCACTTAGACAATC<br>R: TGCTTAGGTNACCTTAAGCATAATCTGGAACAT	*Sal*I<br>*BstE*11

Table S3. Composition of media for *in vitro* culture (for 1 L total volume).

Item	MSP	MSO	MSS	MSR	HMS
MS powder	4.4 g	4.4 g	4.4 g	4.4 g	2.2 g
Casein hydrolysate	300 mg	300 mg	300 mg	100 mg	
Proline	500 mg	500 mg	500 mg		
Sucrose	30 g	30 g	30 g		10 g
Maltose				30 g	
Mannitol		72.8 g			
2.4-D (5 mg/mL)	500 µL	500 µL	500 µL		
Adjust pH to 5.8					
Phytagel	5 g	3 g	5 g	4 g	3 g +2 g agar
Autoclave at 121°C for 20 min					
BAP				3 mg	
NAA				0.5 mg	
Hygromycin			1 mg	1 mg	1 mg

Abbreviations: MS = Murashige & Skoog medium with vitamins, MSP = medium for callus induction, MSO = osmoticum medium prior to bombardment, MSS = selection and callus proliferation medium, MSR = plant regeneration medium, HMS = rooting medium, 2,4-D = 2,4-dichlorophenoxyacetic acid, BAP = 6-benzylaminopurine (systematic name *N*-(phenylmethyl)-7*H*-purin-6-amine), NAA = 1-naphthaleneacetic acid.
